# Design of Temperature Insurance Index and Risk Zonation for Single-Season Rice in Response to High-Temperature and Low-Temperature Damage: A Case Study of Jiangsu Province, China

**DOI:** 10.3390/ijerph16071187

**Published:** 2019-04-02

**Authors:** Ji Guo, Jiajia Jin, Yinshan Tang, Xianhua Wu

**Affiliations:** 1 School of Economics and Management, Shanghai Maritime University, Shanghai 201306, China; 185391@shmtu.edu.cn (J.G.); 15605192693@163.com (J.J.); 2 Collaborative Innovation Center on Climate and Meteorological Disasters, Nanjing University of Information Science & Technology, Nanjing 210044, China; 3 Business Informatics Systems and Accounting, Henley Business School, University of Reading, Reading RG6 6UD, UK; y.tang@henley.ac.uk

**Keywords:** single-season rice, agricultural weather index-based insurance, premium rate determination, Jiangsu Province

## Abstract

Disaster insurance is an important tool for achieving sustainable development in modern agriculture. However, in China, the design of such insurance indexes is far from sufficient. In this paper, the single-season rice in Jiangsu Province of China is taken as an example to design the high-temperature damage index in summer and the low-temperature damage index in autumn to construct the formula calculating the weather output and single-season rice yield reduction. The daily highest, lowest and average temperatures between 1999 and 2015 are selected as main variables for the temperature disaster index to quantitatively analyze the relationship between the temperature index and the yield reduction rate of the single-season rice. The temperature disaster index can be put into the relevant model to obtain the yield reduction rate of the year and determine whether to pay the indemnity. Then, the burn analysis is used to determine the insurance premium rate for all cities in Jiangsu Province under four-level deductibles, and the insurance premium rate can be used for the risk division of the Province. The research provides some insights for the design of agricultural weather insurance products, and the empirical results provide a reference for the design of similar single-season rice temperature index insurance products.

## 1. Introduction

Climate warming increases the possibility of weather disasters for agriculture [1]. It is a recurring subject of human society, particularly how agriculture draws on advantages and avoids weather disasters [2]. China is located in the middle latitudes of the northern hemisphere, with various and complex weather system and frequent weather related disasters. Over the past 30 years, weather disasters such as cold springs, droughts and floods, cold winds in autumn, and frost have affected one-third of China’s arable land, causing significant economic losses [3]. Thus, how to avoid losses and maintain farmers’ incomes with weather insurance for agricultural products draws considerable attention from the government, academia and the public [4].

“Good harvests in Jiangsu and Zhejiang, enough food for old and young” [5]. Jiangsu Province has been a traditional rice producer, with the largest area of *japonica* rice plantation in southern China. The *japonica* rice in Jiangsu is dominated by single-season rice [6], which is susceptible to abnormal temperature during the growing season. During the flowering period of single-season rice, more than two days of temperature greater than 35 °C may cause up to 25 percent of empty seed [7]. The temperature above 35 °C for single-season rice in the booting, heading and flowering stages may lead to a sharp decline in photosynthesis and rise in transpiration, resulting in flower and fruit abortion [8]. Low-temperature damage in autumn for single-season rice in the filling and ripening period may delay maturity. Since the 1990s, the frequency of low-temperature damage has increased in Jiangsu. In August and September, many districts experience such damage with an average temperature of less than 20 °C for more than three consecutive days, increasing the chance of empty rice shell at the heading and filling stages. The listed risks justify the development of weather index-based insurance to protect the income of rice farmers in Jiangsu.

Policy-oriented agricultural insurance is effective tools to promote agricultural development, however, policy-based agricultural insurance takes actual disaster losses as the basis for compensation and the operation for compensation is very complicated. In addition, a high level of disagreement exist between insurance company and insurance applicant for check danger, decide loss, manage compensate and etc., which limited the implementation of the insurance business. The deficiency of policy-based agricultural insurance provides market space for the design and application of weather index insurance. The core of weather index agricultural insurance is the design of weather index.

The indemnity for weather index insurance is determined based on the value of the weather index, and the indemnity amount is based on the estimated loss distribution. Once the weather index parameter reaches the trigger value (if the cumulative rainfall falls below a certain threshold), all policyholders of insurance companies will obtain indemnity [9]. According to Gommes and Kayitakire, although the index-based insurance have advantages, such as the design basis risk, spatial basis risk and idiosyncratic basis risk [10], agricultural weather index-based insurance can partly help avoid problems in traditional insurance such as asymmetric information and high transaction costs, and transfer the risk of weather disasters to insurance companies, which is of significance for promotion. However, at present, there are no weather insurance products specifically targeting single-season rice in Jiangsu. To fill this gap, this paper intends to design a single-season rice weather index based on local conditions to effectively transfer agricultural production. It not only enriches the theoretical research of the weather insurance index, but also provides the reference for the practical application of the weather insurance index in Jiangsu and similar regions in the world. The rest of this paper is as follows: the second part is research summary, the third part is research data and methods, the fourth part is results analysis, and the last part is conclusions.

## 2. Research Summary

Scholars (e.g., [11,12,13,14,15,16,17]) have paid attention to the study of theories and methods of weather index-based insurance design. For example, Skees et al. designed the calculation method of the insurance payout, and divided the difference between the trigger index and the weather index and the lower limit of the trigger index to determine the corresponding rate [11]. Zanini et al. fitted the detrended per unit yield of soybeans and corn in 26 farms and determined the actuarially fair premium rate in different regions with different statistical models [12]. Turvey et al. eliminated the factors that affected crop yield such as altitude and latitude and longitude, and only considered the weather index that had a major impact on Canadian grape production. Turvey et al. used the Monte Carlo model to estimate the premium rate of grapes [13]. Clarke et al. completed the design and pricing of the weather index-based insurance product portfolio with the Bayesian model, and improved the accuracy of the model [14]. Norton et al. found that the spatial-temporal characteristics of the area affected the accuracy of their weather index, resulting in an increased risk of basis error [15]. Miranda et al. designed a new type of insurance to divide a weather index-based insurance contract into multiple equal parts. The standard unit of insurance has the same rate payment time, and farmers can choose to purchase the insurance according to the area of the crop [16]. Castaneda et al. assessed the insurance coverage and claims in rainfall-related risks in processing tomato in Western Spain [17]. From the above reviews, it can be observed that most of the authors used historical distributions to estimate the models and calculate actuarially fair premiums. However, in this research, the burn analysis is used to determine the insurance premium rate due to data restriction.

In China, scholars (e.g., [18,19,20,21,22,23]) have mainly studied the impact of single or integrated weather disaster factors such as continuous rain, drought, high-temperature damage, low-temperature damage, and strong wind on per unit yield, and designed different types of weather index-based insurance products according to local conditions. For example, Wang and Zhang used cotton per unit yield data from three counties in Xinjiang to fit different distribution models and obtained different premium rates. They concluded that only the optimal per unit yield risk distribution model could determine the relatively accurate pure rate [18]. Wu et al. considered many weather factors to establish the rice yield reduction model and designed the premiums of three risk areas under different deductibles [19]. Lu obtained the relations between weather index and grain yield through panel data modeling. With the probability distribution of the disaster, he designed the grain weather index insurance contract [20]. Zhou determined that low-temperature damage was the main weather disaster for apples in Shandong Province. After the yield and trend output were separated with a reasonable low-temperature damage index, the linear relation between Qixia City’s annual per unit yield and low-temperature damage index was determined, and the risk zoning and rate determination were finally determined [21]. From our research perspective, few papers studied the agricultural weather disaster insurance of single-season rice of Jiangsu. To fill this gap, this paper will therefore investigate the actuarially fair premium rate under different deductibles, and then classify different weather disaster risk regions.

The details of the design, application and marketing of index-based insurance varies between countries [22]. Examples include Thailand’s Coffee Rainfall Insurance, Rwanda’s Tomato Weather Index-based Insurance, Ethiopia’s Grain Rainfall Index-based Insurance, and Canadian Forage Index-based Insurance. After Shanghai introduced the first agricultural weather index-based insurance in 2007, the Watermelon Plum Rain Index-based Insurance, other provinces began to promote weather index-based insurance. For instance, in 2008, Anhui Province developed a drought-flood weather index-based insurance for rice in vulnerable rural areas; in 2009, Shaanxi Province carried out a weather index-based insurance for apples; Fujian Province launched a pilot typhoon disaster weather index-based insurance in 2010; in 2016, insurance companies launched “Wind Index-based Insurance for Crops” purchased through Alipay. Overall, as most of China’s weather index-based insurance models adapted from foreign countries, so it is necessary to adjust them according to local conditions. As a national grain-producing province, Jiangsu has not yet promoted the use of agricultural weather index-based insurance, and has not applied the temperature weather index-based insurance for rice [23]. This paper thus establishes the summer high-temperature damage index and the autumn low-temperature damage index to quantify the relationship between the single-season rice temperature index and the yield reduction rate. And then, the single-season rice weather insurance index is constructed.

## 3. Research Data and Methods

### 3.1. Data Sources

**Figure 1:**
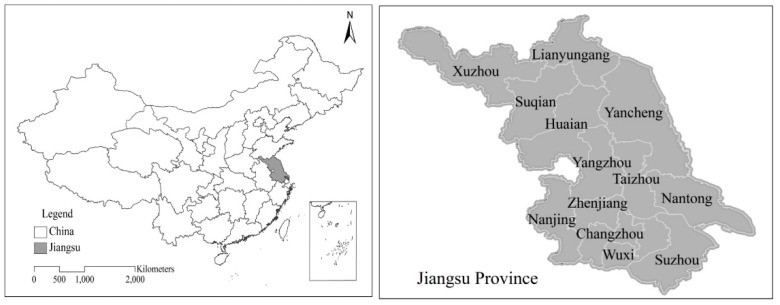
Location of Jiangsu Province in China (of China’s 9.6 million km^2^, Jiangsu covers 107,200 km^2^. The area of 13 cities in Jiangsu are as follows: Nanjing with a total area of 6587 km^2^, Wuxi with 4628 km^2^, Xuzhou with 11,258 km^2^, Changzhou with 4385 km^2^, Nantong with 8544 km^2^, Suzhou with 8488.42 km^2^, Lianyungang with 7615 km^2^, Huaian with 10,072 km^2^, Yancheng with 17,000 km^2^, Yangzhou with 6591.21 km^2^, Zhenjiang with 3848 km^2^, Taizhou with 5787.26 km^2^, Suqian with 8555 km^2^).

The weather data mainly includes the daily minimum, maximum, and average temperature of eight weather stations in Jiangsu from 1999 to 2015. The data comes from the National Meteorological Information Center and the Meteorological Observatory of Nanjing University of Information Science and Technology. The total production and area of single-season rice come from the statistical yearbooks of Jiangsu (This is the data set available at the present, the statistics of prefecture level cities are unavaliable). Figure 1 shows the location of Jiangsu Province in China.

### 3.2. Determination of Weather Production and Yield Reduction

In general, crop yields consist of trend output, weather output, and random error terms. Trend output is determined by factors such as productivity and agricultural technology [20]. Weather output is affected by weather factors such as drought, flood, rainfall, and temperature. Random error terms are caused by sporadic incidents such as insect pests. According to the actual production data of eight cities like Nanjing in Jiangsu from 1999 to 2015, the yield data is processed with the 5-year moving average method, and the single-season rice yield series are divided into trend yield and weather yield, as shown in the following formula:

(1) $$Y=Y_{t}+Y_{w}+\varepsilon$$

Y represents the actual production, $Y_{t}$ the trend output, and $Y_{w}$ the weather output. ε is the random error terms, which are generally omitted in the calculation. The actual output Y minus the trend output $Y_{t}$ is the weather output *Y_w_*. When $Y_{w}$ is greater than 0, it means that the weather conditions are beneficial to increase the yield of single-season rice. When $Y_{w}$ is less than 0, it means that the current weather conditions will reduce single-season rice production.

The ratio of the difference between the actual output of each city and its trend production to the trend output is defined as the relative weather output $S_{i}$:

(2) $$S_{i}=\frac{Y_{i}-Y_{it}}{Y_{it}}\times 100\%,\;i,t=1,2,\ldots ,n$$

*S_i_* is the relative weather output that is not subject to productivity and time constraints. When *S_i_* is less than 0, it means that the single-season rice yield reduces due to the weather, and its absolute value is defined as the yield reduction rate *x_i_*:

(3) $$x_{i}=\left\{ \begin{array}{ll} \left|S_{i}\right| & ,S_{i}<0\\ 0 & ,S_{i}>0 \end{array}\right.,i=1,2,\ldots ,n$$

### 3.3. Weather Index Selection and Design

#### 3.3.1. Single-Season Rice High-Temperature Damage Index

High-temperature damage is a common weather disaster in Jiangsu in summer [24]. Since the 1970s, the frequency of high-temperature damage in Jiangsu has risen steadily. It concentrates in July and August when the rice are in jointing and flowering stages. Continuously rising temperature above 35 °C may lead to a decrease in stamen pollen activity, which inhibits fertilization, affects grouting, and increases the empty seed rate. This is the phenomenon of heat-forced maturity [24].

According to the data of 57 weather stations in Jiangsu, since the 1980s, the frequency of daily maximum temperatures greater than 35 °C for three or more consecutive days has been increasing. It peaked in the 1990s, with an average of 1.57 times per year. From the spatial distribution, the occurrence of extreme high-temperature in the south of Huaihe River is greater than the north of the river. The daily maximum temperature higher than 35 °C for three consecutive days or more in the north of the river is least in frequency. The average frequency in Ganyu and Dongtai of Lianyungang is the lowest, and the average annual number in Xuzhou, Huai’an, and Nantong is about 0.9. Nanjing, Suzhou, Wuxi and Changzhou have averages of over two times per year [25]. Qiang concluded that 35 °C is the critical temperature to mark the high-temperature damage [26]. In addition, when the daily average temperature is higher than 30 °C and the maximum is greater than 35 °C, a 3–4 day duration causes slight high-temperature damage, 5–7 day duration causes medium high-temperature damage, and longer than 8 day causes severe high-temperature damage. The cumulative value of the high-temperature difference is used as an evaluation index of high-temperature damage:

(4) $$HT=\left\{ \begin{array}{ll} 0 & ,else\\ \sum_{i=d1}^{d2}\Delta T=\sum_{i=d1}^{d2}(T_{i}-35) & ,T_{i}\geq 35,T\geq 30 \end{array}\right.$$

The onset time for high-temperature damage in Jiangsu is set at July 15 and the end time is set at August 19. *T_i_* is the maximum temperature at the start date, and once the threshold value is triggered during the statistical period, *T_i_* will be put in the index *HT*.

#### 3.3.2. Single-Season Rice Low-Temperature Damage Index

An abnormally low-temperature during the reproductive period may destroy the physiological structure of the crops, preventing the timely opening of anthers, increasing empty seeds and reducing production [27]. Such low-temperature damage is called “sterile-type low-temperature damage”, which often occurs in September. For the single-season rice in Jiangsu, only the low-temperature damage in September should be considered. At this time, the critical temperature for single-season rice flowering and maturation is 20 °C. Below this temperature, the number of tillers will be reduced and even though the pollen grains can be fertilized normally, they cannot develop into normal full grains.

According to the Technical specifications for evaluation of rice cold damage [QX/T 182-2013], one of industry standards of the China Meteorological Administration [28], an average daily temperature lower than 20 °C for 3 to 4 days causes slight low-temperature damage, 5 to 6 days causes moderate low-temperature damage, and longer than 7 days causes severe damage. Since the 1980s, the average number of slight low-temperature damage in the single-season rice growing season in Jiangsu is between 0 and 11, with the maximum number happed in 1988. Severe low-temperature damage reached 7 times in 1997. In terms of spatial distribution, the southern areas such as Nanjing, Suzhou, Changzhou, and Wuxi have more low-temperature damage and less serious low-temperature damage than the northern areas, while the northern areas such as Xuzhou, Lianyungang, and Yancheng have more severe low-temperature damages than the southern areas.

Since the grain heading and filling period of single-season rice in Jiangsu is from 21 August to 27 September, given the influence of the low-temperature damage index on the yield, the temperature below 20 °C during the statistical period is recorded as a time of low-temperature damage. The formula for calculating the low-temperature damage index in autumn for single-season rice is as follows:

(5) $$LT=\left\{ \begin{array}{ll} 0 & ,else\\ \sum_{j=1}^{n}(T_{0}-T_{j}) & ,T_{j}\leq 20,j=1,2,\ldots ,n \end{array}\right.$$

In above formula, *T*_0_ is the minimum temperature at the stage of the grain heading and filling; *T_j_* the daily average temperature below the lower limit; *n* the number of days below 20 °C.

### 3.4. Design of Single-Season Rice Temperature Index-Based Insurance in Jiangsu

#### 3.4.1. Relations between the Yield Reduction and Weather Disaster Index in Single-Season Rice

In order to analyze the influence of high-temperature damage index and low-temperature damage index on yield reduction rate, the yield reduction rate is used as explained variable, and high-temperature damage index and low-temperature damage index are used as explanatory variables; For the curve effect of these two explanatory variables on dependent variables, the quadratic terms of high-temperature damage index and low-temperature damage index are taken as explanatory variables to construct the following regression equation:

(6) $$S_{i}=F(HT,LT,HT^{2},LT^{2})=a+\sum_{i=1}^{n}b_{i}y_{i}+\mu_{i},\;i=1,2,\ldots ,n$$

In this formula, *S_i_* is the yield reduction rate of single-season early rice in 8 cities in Jiangsu, *HT* is the high-temperature damage index, *LT* is the low-temperature damage index, *a* and *b_i_* are regression coefficient, and *y_i_* is the weather disaster index.

#### 3.4.2. Determination Method of Pure Insurance Premium Rate

The actuarially fair premium rate of crop insurance is equal to loss expectancy, which is the value of actuarially fair premium minus the amount of insurance [29]. The formula for actuarially fair premium rate of single-season rice index insurance is:

(7) $$R_{c}=\frac{E[loss]}{\lambda \mu }=E[loss]$$

*λ* is the index insurance scope of coverage of crops, *μ* the expected per unit yield, and *E*[*loss*] represents the expected loss of single-season rice. According to the Jiangsu’s policy-oriented agricultural insurance pilot program, both *λ* and *μ* can be assumed as 100% [30].

In weather index insurance products, the calculation formula of the single crop rice insurance index is:

(8) $$W=\left\{ \begin{array}{l} 0,\;x\leq x_{c}\\ x,\;x>x_{c} \end{array}\right.$$

In the formula, *W* is the single-season rice insurance index in a certain region of Jiangsu, *x_c_* the deductible of the region and *x* the yield reduction rate in an area caused by weather disasters. By establishing a model of single-season rice and temperature disasters, the corresponding temperature index can be found at the end of the growth period of single-season rice. Then the single-season rice yield reduction rate can be calculated through the temperature index. Based on the regional deductible, whether to implement the indemnity will be determined. The single-season rice insurance index is shown in formula (9), and the calculation formula for pure rice premium of single-season rice under different deductibles is as follows:

(9) $$P_{c}=R_{c}\times Q$$

*R_c_* is the actuarially fair premium rate for different deductibles, and *Q* the insurance amount. According to Jiangsu’s policy-oriented agricultural insurance [25], the single-season rice insurance is recommended with the amount of CNY 6000 per Ha in this paper, namely, *Q* = 6000 CNY/Ha.

The premium rate has the following formula:

(10) $$R_{g}=R_{c}*(1+r_{s})(1+r_{p})(1+r_{b})$$

$R_{g}$ represents the gross rate, *r_s_* the safety coefficient, *r_p_* the profit rate, and *r_b_* the operating cost coefficient. Except the actuarially fair premium rate, the formula is determined by the regional risk and the operating conditions of the insurance company. The following discussion focuses on the determination of the actuarially fair premium rate.

The premium rate of the weather index is determined by the index model method and the burn analysis [31,32]. The index model method uses a distribution to fit historical payouts and estimates the model parameters, thereby calculating actuarially fair premiums. The per unit yield distribution model is a common derivation method for an actuarially fair premium rate. However, it is difficult to verify per unit yield distribution function of each region, and it is error-prone. The burn analysis is used to determine the insurance premium rate. This method assumes that the expected loss rate in the future and the past loss distribution are the same (According to Jewson and Brix (2005), burn analysis uses historical data to evaluate the value of derivative contracts. When use the burn analysis, the basic hypothesis includes: (1) the time series data of temperature is stable; (2) the data of each year are independent and subject to the same distribution. Therefore, the burn analysis is regarded as a simplified pricing method, which can estimate price conveniently [32]). The expected loss rate is estimated through historical production data:

(11) $$S_{i}=\frac{1}{n}\sum \frac{Y-Y_{t}}{Y_{t}}\;,Y-Y_{t}<0;i,t=1,2,\ldots ,n$$

(12) $$R_{c}=E[loss]=\frac{1}{n}\sum \left|S_{i}\right|,S_{i}<0,i=1,2,\ldots ,n$$

*E*[*loss*] is the expected yield reduction rate of each city, n the length of time series, and *R_c_* = *E*[*loss*] is the actuarially fair premium rate of each city in Jiangsu. According to above formula, the yield reduction rate of the corresponding year in each city, namely, the indemnity rate can be obtained:

(13) $$L_{t}=\left\{ \begin{array}{l} 0,x\leq x_{c}\\ x,x>x_{c} \end{array}\right.$$

In this case, the average indemnity rate of 17 years is:

(14) $$L=\frac{1}{17}\sum_{t=1}^{17}L_{t}$$

In the past, the determination of the premium rate was usually made by the average rate of indemnity, because the average loss rate of crops has sound stability, and the fluctuation of the loss rate is small, which is a relatively good estimate of the actuarially fair premium rate. However, the insurance indemnity and crop loss in different regions are different, and the average loss rate fluctuates. Therefore, the determination of actuarially fair premium is improved in this paper:

(15) $$R_{c}=L(1+\delta )$$

(16) $$\delta =\frac{\sigma }{\bar{L}}$$

In the formula, σ and $\bar{L}$ are the standard deviation and the mean of L, respectively. δ is the coefficient of variation, which reflects the degree of dispersion of the average yield reduction rate in several years.

## 4. Results and Analysis

### 4.1. Regression Analysis

Taking the year-by-year yield reduction rate as the dependent variable, and the high-temperature damage and the low-temperature damage as the independent variables to establish the regression model for the single-season rice yield reduction rate and temperature index in each city of Jiangsu. Using R and SPSS for stepwise regression, the regression models of the eight cities (Due to the lack of data from five cities in Taizhou, Suqian, Huai’an, Yangzhou and Zhenjiang, only regression analysis for eight cities including Nanjing and Suzhou in Jiangsu Province is conducted.) are significant overall (the *P*-value of the F-test value is less than 5%) (In model (6), the potential multicollinearity, heteroscedasticity and autocorrelation of variables are tested by Pearson correlation analysis, Whiter test and Durbin- Watson test, respectively. We found the multicollinearities between independent variables are not existed. Heteroscedasticity or autocorrelation of variables are eliminated through Generalized Least Square (GLS). Limited to space, the results are not reported here.). The effect of temperature index on single-season rice yield in Jiangsu is significant at a 10% confidence level. The regression correlation coefficient of each city and the *P* value of the correlation index are shown in the Table 1 below. The single-season rice yield reduction in Xuzhou, Yancheng, Changzhou, and Suzhou has a significant relation with low-temperature damage. The weather disasters in the southern region are mainly sight low-temperature damage. In other words, the single-season rice yield reduction rate in Lianyungang, Nanjing and Nantong has a significant relation with high-temperature damage and low-temperature damage. See the table below for detail.

### 4.2. Analysis on the Pure Insurance Premium Rate of Cities in Jiangsu under the Deductibles at All Levels

In order to fully calculate the actuarially fair premium rates for cities in Jiangsu, the actuarially fair premium rates for deductibles of 2.5%, 5%, 7.5%, and 10%, respectively, will be calculated below. According to the regression results and the actual production of single-season rice in Jiangsu, the safety surcharge rate is set as 20%, the profit rate 5%, and the operating cost surcharge rate 15%, then the gross insurance premium rate = actuarially fair premium rate × 1.45. The Kriging interpolation method and ArcGIS software mapping are used to intuitively describe the classification of actuarially fair premium rates under various deductibles for each city in the province, and then different risk areas are divided (Based on the data of seven other prefecture-level cities such as Nanjing and Suzhou in Jiangsu Province, this paper interpolates and estimates the actuarially fair premium rates of the five cities of Taizhou, Suqian, Huai’an, Yangzhou and Zhenjiang [31]) (Table 2).

**Figure 2:**
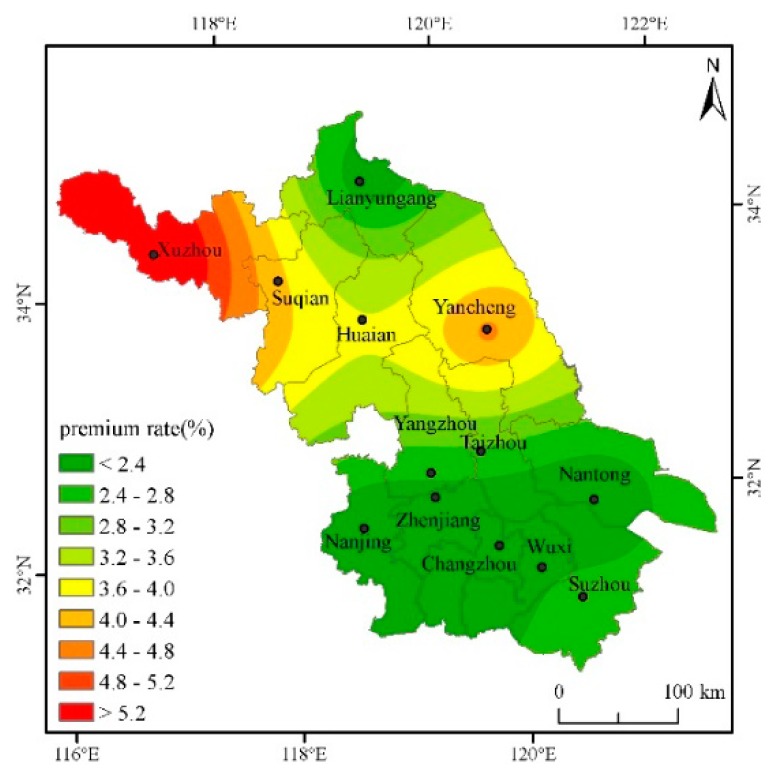
Distribution of actuarially fair premium rate for each city when the deductible amount is 2.5%.

From Figure 2, with a 2.5% deductible, the province’s single-season rice actuarially fair premium rate is between 1.902% and 6.180%. The rates in Xuzhou and Yancheng are high, more than 4%; the rates in southeastern Jiangsu are low. The rates in the central region are between those of the north and the south.

**Figure 3:**
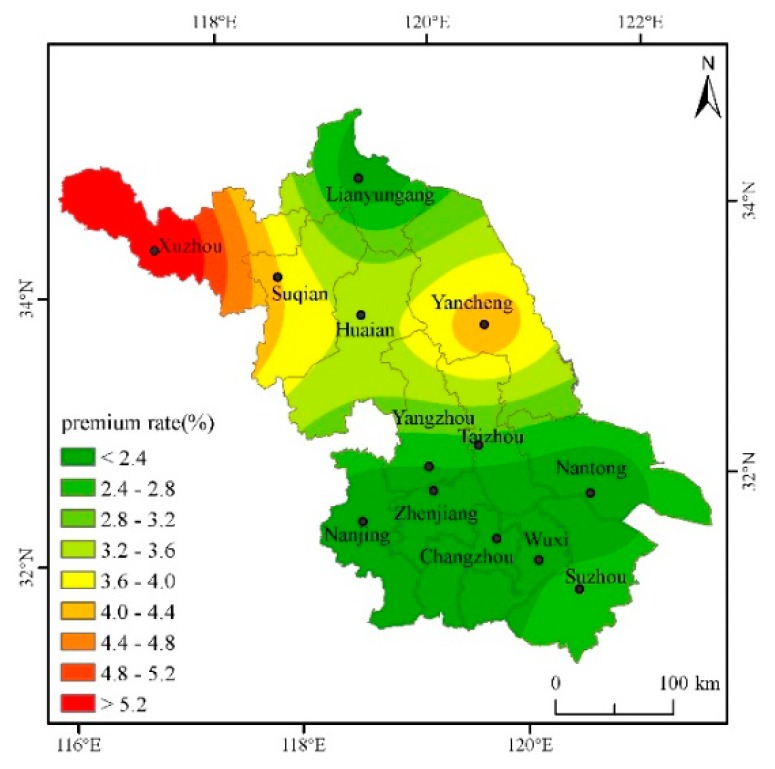
Distribution of actuarially fair premium rate for each city when the deductible amount is 5%.

From Figure 3, with a 5% deductible, the province’s actuarially fair premium rate is between 1.80% and 6.12%, which is lower than the 2.5% deductible. The actuarially fair premium rate in the southeast region of Jiangsu is still the lowest, lower than 2.7%.

**Figure 4:**
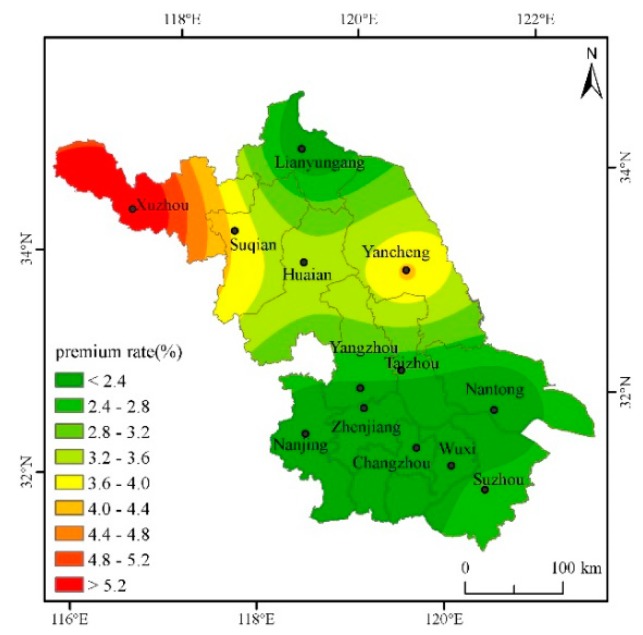
Distribution of actuarially fair premium rate for each city when the deductible amount is 7.5%.

From Figure 4, with a 7.5% deductible, the province’s actuarially fair premium rate continues to fall, from 1.781% to 6.010%. The premium rates in the northeast are still the highest, but it is clear from the figures that the range of high rates has narrowed.

**Figure 5:**
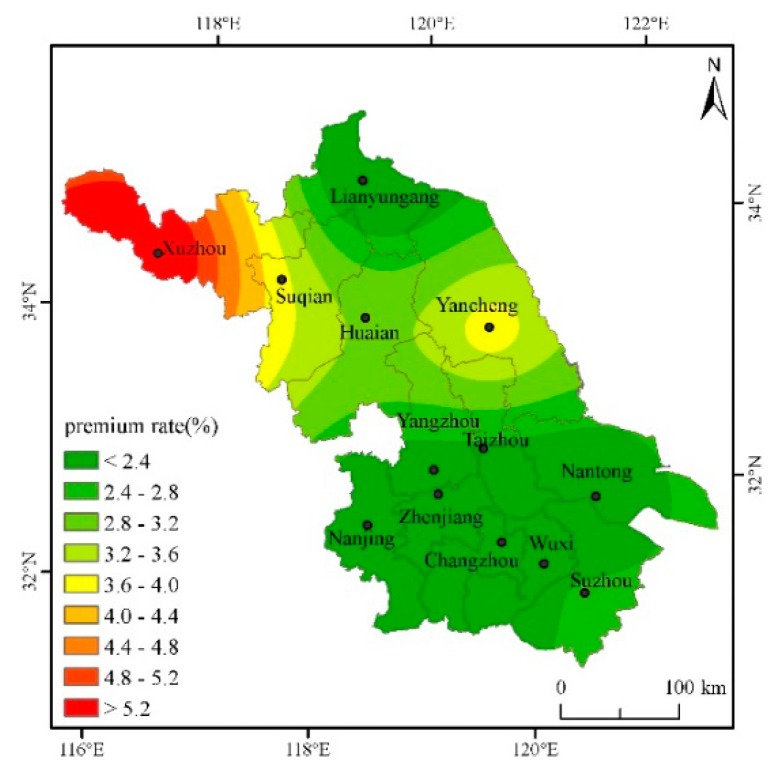
Distribution of actuarially fair premium rate for each city when the deductible amount is 10%.

From Figure 5, under a 10% deductible, the actuarially fair premium rate fluctuates from 1.51% to 6.01%. Only Xuzhou city with an actuarially fair premium rate of more than 4%, in many other cities, the actuarially fair premium rates even drop below 2%.

Through the analysis of the actuarially fair premium rates under different deductibles above, it is found that the risk of yield reduction with the deductibles at all levels in the northwestern region of Jiangsu is higher and the rates are higher than those in other regions, while the rates in the southeastern cities are lower. According to the distribution of actuarially fair premium rates with the deductibles at all levels and the actual conditions of agricultural insurance Jiangsu, different deductibles can be set in different risk regions to reduce the probability of adverse selection and opportunistic behavior.

The single-season rice insurance premium rate is affected by weather disasters, and a greater disaster risk means a greater single-season rice premium rate. On the whole, the risks of high-temperature damage and low-temperature damage are not too high, but the deductible cannot be set to the same in different cities. Although the same deductible is beneficial to the management of insurance companies, the probability of occurrence of risks in each region is entirely different, and it is easy to cause basis risk. It is proposed to divide Jiangsu into two risk areas, where Xuzhou and Yancheng are the first risk area, and Lianyungang, Nanjing, Changzhou, Nantong, Wuxi, and Suzhou are the second category of high risk area. Xuzhou’s rates are the highest with various deductibles. The main reason is that Xuzhou’s low-temperature index is higher than those of other cities. That is, the number of days with an average daily temperature below 20 degrees during the autumn grouting period is the highest, resulting in high yield reduction rates. Yancheng’s actuarially fair premium rate is slightly lower than that of Xuzhou, but it is significantly higher than in other cities. The same deductible of 5% can be set to reduce management costs. For other cities, such as Lianyungang, Nanjing, Changzhou, and Nantong, both the actuarially fair premium rate and the total premium rate are very close. Therefore, in determining the actuarially fair premium rate, the same deductible for the other cities may be set at 2.5% to reduce the insurance company’s management costs.

## 5. Discussion and Conclusions 

### 5.1. Research Conclusion

The single-season rice high-temperature and low-temperature indices of Jiangsu are designed based on daily weather data and single-season rice production data from eight cities from 1999 to 2015. The relations between single-season rice yield reduction rate and temperature index are studied, the actuarially fair premium rates of single-season rice temperature index-based insurance in Jiangsu are determined, the single-season rice temperature index insurance in Jiangsu is designed, and the risk area of single-season rice in Jiangsu is divided. This paper proposes to take Xuzhou City as Risk Area One, and other cities as Risk Area Two. These results can provide a reference for the application and extension of agricultural insurance in Jiangsu or beyond (we sent the results to three officials of the development and reform commission of Jiangsu Province; they thought that this research could improve their work and be of significance). In addition, for the managerial implications, it is necessary to pay attention to the differences of temperatures in different cities and regions. For example, ground sensors and remote sensors can be used to obtain grid data, and then local agricultural meteorological disaster insurance can be formulated.

### 5.2. Research Prospect

The following aspects can be further studied in the future:

(1) Due to the lack of data on single-season rice production in some cities before 2005 in Jiangsu, data from eight cities from 1999 to 2015 is selected for empirical analysis, so the data is incomplete. In addition, the lack of county-level city data makes the actuarially fair premium rate only determined based on prefecture-level cities, so there is still a certain basis risk.
(2) The weather output is separated through the moving average method, losing some production data. In the future, better models can be selected by comparing other models such as the Autoregressive Integrated Moving Average (ARIMA) model and the linear moving average method.
(3) Many factors are affecting the fertility of single-season rice. In this paper, only the high-temperature damage and low-temperature damage indexes are included in the model, which can be further enriched in the future.

## Figures and Tables

**Table 1:** Regression Report on Yield Reduction Rate of Single-Season Rice and Temperature Index in Jiangsu.

City	High-Temperature Damage Index	Square of High-Temperature Index	Low-Temperature Damage Index	Square of Low-Temperature Damage Index	Adjusted R Square
Xuzhou	−0.00468	−0.00361	−0.041866 **	−0.003416 **	0.539
	(0.165)	(0.258)	(0.026)	(0.024)	
Yancheng	−0.00033	−0.00842 **	−0.008343 **	−0.000039 *	0.543
	(0.241)	(0.186)	(0.048)	(0.061)	
Lianyungang	−0.022362 **	−0.004589 **	0.003008 ***	−0.000051**	0.492
	(0.047)	(0.037)	(0.009)	(0.004)	
Nanjing	0.000712 ***	−0.000873 ***	−0.009874 **	−0.000401 **	0.506
	(0.006)	(0.002)	(0.049)	(0.059)	
Nantong	0.003358 **	−0.000108 **	0.006685 ***	−0.000381 ***	0.450
	(0.015)	(0.046)	(0.001)	(0.005)	
Changzhou	0.0273	−0.000912 **	0.010007 *	−0.000771 ***	0.422
	(0.155)	(0.037)	(0.055)	(0.002)	
Wuxi	−0.002780 **	−0.039	−0.013001 **	−0.042	0.616
	(0.049)	(0.127)	(0.042)	(0.131)	
Suzhou	−0.00745	−0.00631	−0.005343 **	−0.0000389 **	0.639
	(0.26)	(0.142)	(0.024)	(0.024)	

Note: (1) In the brackets are the *p* value; (2) * indicates the significance level at 10%, ** the significance level at 5%, and *** the significance level at 1% Jiangsu.

**Table 2:** Premium rates of each city under different deductibles.

City	2.5% Deductible		5% Deductible		7.5% Deductible		10% Deductible	
	Actuarially Fair Premium Rate (%)	Actuarially Fair Premium (CNY)	Actuarially Fair Premium Rate (%)	Actuarially Fair Premium (CNY)	Actuarially Fair Premium Rate (%)	Actuarially Fair Premium (CNY)	Actuarially Fair Premium Rate (%)	Actuarially Fair Premium (CNY)
Xuzhou	6.18	370.80	6.12	367.20	6.01	360.60	6.01	360.60
Yancheng	4.53	271.80	4.41	264.60	4.12	247.20	3.91	234.60
Lianyungang	2.04	122.40	1.98	118.80	1.89	113.40	1.51	90.60
Nanjing	1.90	114.12	1.80	108.12	1.78	106.86	1.53	92.04
Changzhou	1.91	114.66	1.89	113.16	1.82	109.38	1.59	95.10
Nantong	1.85	111.06	1.85	111.00	1.85	110.94	1.72	103.26
Wuxi	2.22	133.38	2.21	132.84	2.01	120.72	2.00	120.06
Suzhou	2.71	162.54	2.70	161.88	2.68	161.04	2.55	152.82

## References

[1] Field C.B., Barros V., Stocker T.F., Qin D., Dokken D.J., Ebi K.L., Mastrandrea M.D., Mach K.J., Plattner G.-K., Allen S.K. (2012). Managing the Risks of Extreme Events and Disasters to Advance Climate Change Adaptation: IPCC Special Report on Working Groups I and II of the Intergovernmental Panel on Climate Change. J. Clin. Endocr. Metab..

[2] Lesk C., Rowhani P., Ramankutty N. (2016). Influence of extreme weather disasters on global crop production. Nature.

[3] Huang R.H. (2006). Progresses in Research on the Formation Mechanism and Prediction Theory of Severe Climatic Disasters in China. Adv. Earth Sci..

[4] Zhang J., Zhang S., Cheng M., Jiang H., Zhang X., Peng C., Lu X., Zhang M., Jin J. (2018). Effect of Drought on Agronomic Traits of Rice and Wheat: A Meta-Analysis. Int. J. Environ. Res. Public Health.

[5] Wang S.Z., Zhu G.Z. (1997). Records of Song Poems 100.

[6] Zuo J., Wang X.J., Guo Y.L., Kong H., Zhou X., Huang Q.X., Jia R.Z., Xu L., Guo A.P. (2014). Weed Species in A Transgenic Rice Field at the Plant Breeding Base of Hainan Province. Weed Sci..

[7] Xu H.B., Wang G.M., Wei M., Zhou W.B. (2001). Correlation Analysis of the Characters of Pollen Grains and Seed-setting of Rice under High Temperature Stress. J. Southwest Agric. Univ..

[8] Luo L.H., Liu G.H., Xiao Y.H., Tang W.B., Chen L.Y. (2005). Influences of High-Temperature Stress on the Fertility of Pollen, Spikelet and Grain-Weigh in Rice. J. Hunan Agric.Univ..

[9] World Bank (2013). Weather Index Insurance for Agriculture: Guidance for Development Practitioners.

[10] Leblois A., Quirion P., Sultan B. (2014). Price vs. weather shock hedging for cash crops: Ex ante evaluation for cotton producers in Cameroon. Ecol. Econ..

[11] Skees J.R., Hazell P.B.R., Miranda M. (1999). New Approaches to Crop Yield Insurance in Developing Countries. Eptd Discuss. Pap..

[12] Zanini F.C., Sherrick B.J., Schnitkey G.D., Lrwin S.H. (2004). Crop Insurance Valuation under Alternative Yield Distributions. Am. J. Agric. Econ..

[13] Turvey C.G., Weersink A. (2005). Pricing Weather Insurance with a Random Strike Price: An Application to the Ontario Ice Wine Harvest. Am. J. Agric. Econ..

[14] Clarke D.J., Clarke D., Mahul O., Verma N. (2012). Index Based Crop Insurance Product Design and Ratemaking: The Case of Modified Nais in India.

[15] Norton M.T., Turvey C., Osgood D. (2012). Quantifying Spatial Basis Risk for Weather Index Insurance. J. Credit Risk..

[16] Miranda M., Skees J.R., Hazell P. (1999). New Approaches to Public/Private Crop Yield Insurance.

[17] Castaneda V.A., Barrios L., Garrido A. (2014). Assessment of Insurance Coverage and Claims in Rainfall Related Risks in Processing Tomato in Western Spain. Eur J Agron..

[18] Wang K., Zhang Q. (2010). Influence of Flexible Crop Yield Distributions on Crop Insurance Premium Rate: A Case Study on Cotton Insurance in Three Counties of Xinjiang Province. J. China Agric. Univ..

[19] Wu L.H., Lou W.P., Yao Y.P., Mao Y.D., Su G.L. (2010). Design of Products for Rice Agro-Meteorological Index Insurance: A case in Zhejiang Province. Sci. Agric. Sin..

[20] Lu P. (2010). Design of Weather Index Crop Insurance Contract for Northeast China.

[21] Wu X.H., Xu Z., Liu H., Guo J., Zhou L. (2019). What are the impacts of tropical cyclones on employment?—An Analysis Based on Meta-regression. Wea. Climate and Soc..

[22] Wu X.H., Cao Y.R., Xiao Y., Guo J. (2018). Finding of Urban Rainstorm and Waterlogging Disasters Based on Microblogging Data and the Location-routing Problem Model of Urban Emergency Logistics. Ann. Oper. Res..

[23] Bai L., Duan D.X., Wan Z. (2014). Evaluating Production Risk in Rice Production of Guangdong Province. Southwest China. J. Agric. SCI-Camb..

[24] Zhang F.F. (2012). The Spatial and Temperature Distribution of Rice Heat Injury Hubei Province. J. Huazhong Agric. Univ..

[25] You Z.X. (2008). Research on System of Agricultural Insurance in Jiangsu Province.

[26] Qiang H.T. (2011). Study on High Temperature Induced Heat Damage and its Impacts on Rice Production in Jiangsu Province.

[27] Meng Z.H., Wang Y.J., Sun Z.S., Meng Q.X., Liu Y.W., Lei C.G., Zhang J.L. (2005). The Research of Rice Sterile-type Chilling Injury and Irrigation Technique. Chin. Agric. Sci. Bull..

[28] China Meteorological Administration (2013). Technical Specifications for Evaluation of Rice Cold Damage [QX/T 182-2013].

[29] Ker A.P., Goodwin B.K.G. (2000). Nonparametric estimation of crop insurance rates revisited. Am. J. Agric. Econ..

[30] Lou W.P., Ji Z.W., Qiu X.F. (2011). Design of Tea Frost Weather Index Insurance. Chin. J. Nat. Resour..

[31] Guo J., Zhou L., Wu X.H. (2018). Tendency of Embodied Carbon Change in the Export Trade of Chinese Manufacturing Industry from 2000 to 2015 and Its Driving Factors. Sustainability.

[32] Jewson S., Brix A. (2005). Weather Derivative Valuation: The Meteorological, Statistical, Financial and Mathematical Foundations.

